# Risk of Active Tuberculosis in HIV-Infected Patients in Taiwan with Free Access to HIV Care and a Positive T-Spot.TB Test

**DOI:** 10.1371/journal.pone.0125260

**Published:** 2015-05-04

**Authors:** Hsin-Yun Sun, Po-Ren Hsueh, Wen-Chun Liu, Yi-Ching Su, Sui-Yuan Chang, Chien-Ching Hung, Shan-Chwen Chang

**Affiliations:** 1 Department of Internal Medicine, National Taiwan University Hospital and National Taiwan University College of Medicine, Taipei, Taiwan; 2 Department of Laboratory Medicine, National Taiwan University Hospital and National Taiwan University College of Medicine, Taipei, Taiwan; 3 Department of Clinical Laboratory Sciences and Medical Biotechnology, National Taiwan University College of Medicine, Taipei, Taiwan; 4 Department of Medical Research, China Medical University Hospital, Taichung, Taiwan; 5 China Medical University, Taichung, Taiwan; National Taiwan University Hospital, TAIWAN

## Abstract

**Background:**

Interferon-gamma release assays (IGRAs) have been used to identify individuals at risk for developing active tuberculosis (TB). However, data regarding the risk of TB development in HIV-infected patients testing positive for IGRAs remain sparse in the era of combination antiretroviral therapy.

**Methods:**

Between 2011 and 2013, 608 HIV-infected patients without active TB undergoing T-Spot.TB testing were enrolled in this prospective observational study at a university hospital designated for HIV care in Taiwan with a declining TB incidence from 72 per 100,000 population in 2005 to 53 per 100,000 population in 2012. All of the subjects were followed until September 30, 2014. The national TB registry was accessed to identify any TB cases among those lost to follow-up.

**Results:**

T-Spot.TB tested negative in 534 patients (87.8%), positive in 64 patients (10.5%), and indeterminate in 10 patients (1.6%). In multivariate analysis, positive T-Spot.TB was significantly associated with older age (adjusted odds ratio [AOR], 1.172 per 10-year increase; 95% confidence interval [CI], 1.022-1.344, P=0.023), past history of TB (AOR, 13.412; 95% CI, 6.106-29.460, P<0.001), and higher CD4 counts at enrollment (AOR, per 50-cell/μl increase, 1.062; 95% CI, 1.017-1.109, P=0.007). Of the 64 patients testing positive for T-Spot.TB, none received isoniazid preventive therapy and all but 5 received combination antiretroviral therapy at the end of follow-up with the latest CD4 count and plasma HIV RNA load being 592.8 cells/μL and 1.85 log_10_ copies/mL, respectively. One patient (1.6%) developed active TB after 167 person-years of follow-up (PYFU), resulting in an incidence rate of 0.599 per 100 PFYU. None of the 534 patients testing negative for T-Spot.TB developed TB after 1380 PYFU, nor did the 24 patients with old TB and positive T-Spot.TB tests develop TB after 62.33 PYFU.

**Conclusions:**

The risk of developing active TB in HIV-infected patients with positive T-Spot.TB receiving combination antiretroviral therapy is low in Taiwan where the national TB program has led to a sustained decrease in TB incidence.

## Introduction

The lifetime risk for active tuberculosis (TB) is estimated to be 5 to 10% for a person with a positive tuberculin skin test (TST) [1], but the risk is much higher in HIV-infected patients, 10% per year [2]. Because interferon-gamma (IFN-γ) plays a pivotal role in regulating cell-mediated immune response against TB, interferon-gamma release assays (IGRAs) were developed to detect *Mycobacterium tuberculosis* infection [3–5]. The Food and Drug Administration (FDA)-approved IGRAs include QuantiFERON-TB (QFT), QuantiFERON-TB Gold test (QFT-G), QuantiFERON-TB Gold In-Tube test (QFT-GIT), and T-Spot.TB [6]. The Centers for Disease Control and Prevention (CDC) recommend IGRAs be used as TST as aids in diagnosing infection with *M*. *tuberculosis*, and IGRA is preferred particularly for persons that have low rates of returning to have TST read or those who have received BCG vaccination [6].

After IGRAs were available, numerous studies have been conducted to further characterize the use of IGRAs. One fixed-effects meta-analysis of 20 studies assessed the performance of IGRAs [7], which showed that the pooled sensitivity was 78% (95% confidence interval [CI], 73% to 82%) for QFT-G, 70% (95% CI, 63% to 78%) for QFT-GIT, and 90% (95% CI, 98% to 100%) for T-Spot.TB [7]. The meta-analysis concluded that T-Spot.TB appeared to be more sensitive than both QuantiFERON tests and TST [7].

Because IGRAs detect a cellular immune response to *M*. *tuberculosis*, IGRAs have reduced sensitivity in immunocompromised patients. In a systematic review of 37 studies including 5736 HIV-infected individuals, the pooled sensitivity estimates were higher for T-Spot.TB (72%; 95% CI, 62%-81%) than for QFT-GIT (61%; 95% CI, 41%-75%), but none of the IGRAs were consistently more sensitive than TST in head-to-head comparisons. Although T-Spot.TB appears to be less affected by immunosuppression than QFT-GIT and TST, the overall differences among these three tests are small or inconclusive [8]. Another systematic review of 38 studies including 6514 patients demonstrated that pooled indeterminate rates were higher in high TB burden settings than in settings of low to intermediate burden and in patients with CD4 lymphocyte counts <200 cells/μL [9].

Given the fact that there is no gold standard for latent TB infection, the only way to confirm the predictive value for progression to TB of TST or IGRAs is the development of active TB in tested persons during follow-up. Two meta-analyses showed different results; one concluded that neither IGRAs nor TST had high accuracy for the prediction of active TB [10], but the other demonstrated that commercial IGRAs had a higher positive as well as negative predictive value for progression to active TB compared with TST [11]. Such discrepancies might result from different inclusion criteria; one included only persons without IPT for analysis [11] while the other did not have such a criterion [10]. Analytic approach adopted also explains such discrepancies; one did not take duration of follow-up into consideration [11], but the other used incidence rates to present the prognostic capability of the IGRAs [10]. Nevertheless, such data in HIV-infected population remain limited [12–16]. Thus, the present study aimed to assess the risk for progression to active TB in HIV-infected patients testing for T-Spot.TB through longitudinal follow-up.

## Materials and Methods

### Setting and Study population

Taiwan has an estimated TB incidence of 53.0 per 100,000 population in the general population in 2012, which has decreased from 72.0 per 100,000 in 2005 with the implementation of the national TB control program aiming to halve TB incidence by 2015 [17]. To make this achievement possible, the national TB control program launched a directly observed treatment short-course program (DOTS) for all identified TB patients, scaling-up of laboratory diagnosis, contact investigation, and treatment of latent TB infection. HIV-infected patients in Taiwan are provided free-of-charge access to HIV care, including combination antiretroviral therapy (cART) that was introduced on April 1, 1997 and monitoring of CD4 lymphocyte counts and plasma HIV RNA loads at designated hospitals around the island. In this study, we enrolled HIV-infected patients aged 20 years or greater who sought HIV care between March 2011 and September 2013 for detection of latent TB infection using T-SPOT.TB assay at the National Taiwan University Hospital. During the study period, isoniazid preventive therapy (IPT) was suggested for patients with positive results, but was initiated at the discretion of primary care physicians after discussion with the patients.

All enrolled patients had regular follow-up as outpatients every 3 to 6 months. Work-up, including chest radiography, acid-fast staining, mycobacterial cultures, and histopathology of the infected sites would be performed if the patients had symptoms or signs highly suspected of TB during the follow-up. The protocol was approved by the Research Ethics Committee of the National Taiwan University Hospital (registration number, 201103060RC) and all patients provided written informed consent prior to enrollment.

### Data collection

A computerized data collection form was used to retrieve the demographic and clinical data of HIV-infected patients from the medical records, including age, gender, risk behaviors for HIV transmission, CD4 count and plasma HIV RNA load, and TB status, active or old. Old TB was considered if the information of the national TB reporting website (https://tb.cdc.gov.tw/slow/CA/LoginByCard.asp) showed that the patient had a history of active TB reported 3 months or greater before the study enrollment, and there were no clinical symptoms and signs of active TB at enrollment. Active TB was considered if the information of the national TB reporting website showed that the patient had a history of active TB reported within 3 months of the study enrollment, and there were clinical symptoms and signs of active TB or culture- or histopathology-confirmed TB within 3 months of the study enrollment. Cross-matching with the national TB reporting website was performed at the end of the follow-up to obtain the data from any patients who were lost to follow-up at this hospital during the study period.

### Laboratory investigations

T-SPOT.TB assay (Oxford Immunotech Ltd., Oxford, UK) was performed to detect latent TB infection in HIV-infected patients, and the results were interpreted according to the CDC’s criteria [6]. HIV infection was diagnosed by detection of anti-HIV antibodies using enzyme-linked immunosorbent assay or particle agglutination (SFD HIV 1/2 PA, Bio-Rad FUJIREBIO, Japan) and confirmed by Western Blot test (MP Diagnostics HIV BLOT 2.2. MP Biomedicals Asia Pacific Pte Ltd, Singapore). Plasma HIV RNA load and CD4 lymphocyte count were quantified by the Cobas Amplicor HIV-1 Monitor Test, version 1.5, (Roche Diagnostics Corporation, Indianapolis, USA) and FACSFlow (BD FACS Calibur, Becton Dickinson, CA), respectively.

### Statistical analysis

All statistical analyses were performed using SPSS software version 17.0 (SPSS Inc., Chicago, IL). Categorical variables were compared using χ^2^ or Fisher’s exact test whereas non-categorical variables were compared using Student’s t-test. A multivariable logistic model was used to estimate the effects of multiple variables on the T-Spot.TB positivity. A backward stepwise selection was used. All variables significant at P<0.20 in the univariate analysis were entered in the model and then removed in a stepwise design if P>0.20. Interactions among the main effects were examined, and the final model was checked with the Hosmer-Lemeshow goodness of fit test. The incidence rate of developing active TB during follow-up period was calculated as the number of episodes of active TB per 100 person-years of follow-up (PYFU). All tests were two-tailed and a *P* value <0.05 was considered significant. The TB status of enrolled HIV-infected patients was followed until the end of the study on September 30, 2014 or death before September 30, 2014, whichever occurred first.

## Results

**Fig 1:**
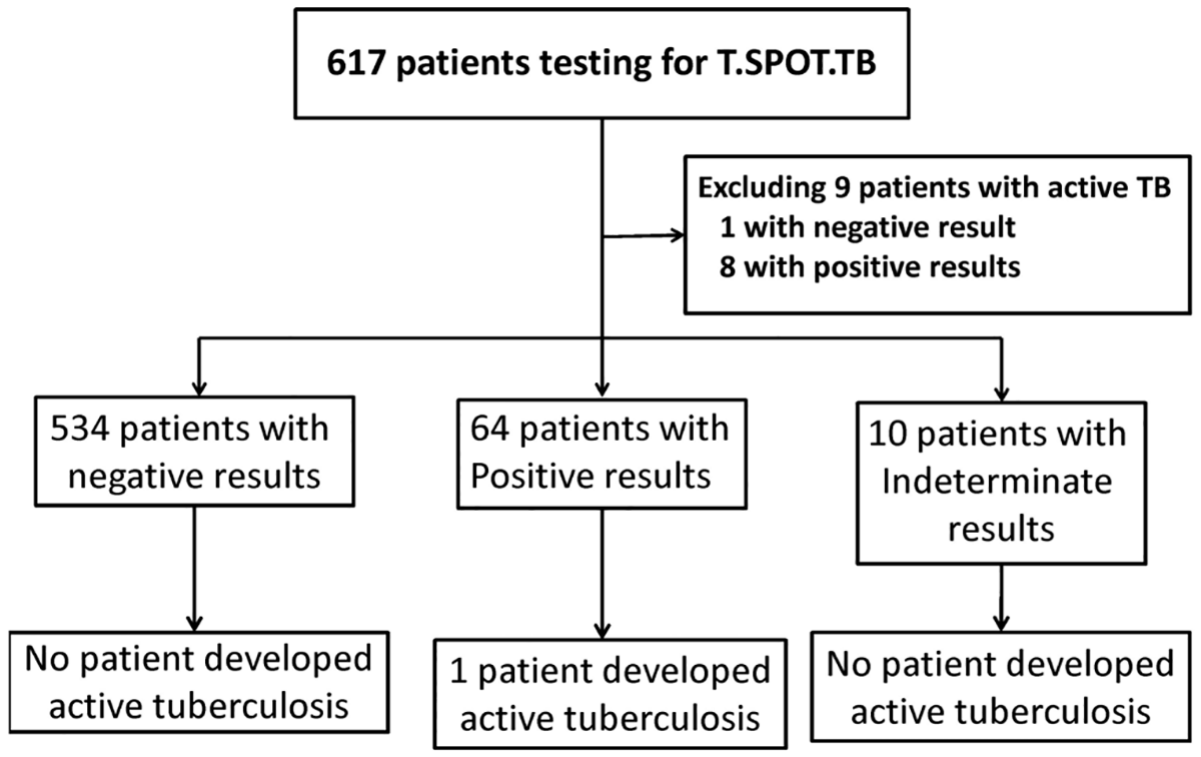
Enrollment and disposition of the study subjects.

Between March 2011 and September 2013, a total of 617 HIV-positive patients were enrolled and followed until their death or September 30, 2014. After exclusion of 9 patients with active TB at enrollment, 543 patients (87.8%) had negative T-Spot.TB results, 64 (10.5%) positive results, and 10 (1.6%) indeterminate results (Fig 1). Of the 608 included study subjects, 81.0% were male homosexuals, 15.7% heterosexuals, 1.5% injecting drug users, and 1.8% others. The mean follow-up duration at the end of the study when TB status was evaluated was 2.57 years, and 15 patients (2.5%) died during the follow-up.

Compared with patients with negative T-Spot.TB results, those with positive T-Spot.TB results had a significantly higher mean (± standard deviation [SD]) CD4 count (578.4 ± 306.5 vs 441.6 ± 295.9 cells/μL) and lower mean (± SD) plasma HIV RNA load (2.35 ± 1.23 vs 2.89 ± 1.50 log_10_ copies/mL) and were significantly older (mean age [± SD], 45 ± 13.1 vs 36.9 ± 10.8 years), less likely to be male (90.6% vs 97.6%), and more likely to have a history of old TB (37.5% vs 3.7%) and to receive cART at the time of T-Spot.TB testing (84.4% vs 61.0%) (all *P* values <0.05) (Table 1). After a mean (± SD) follow-up duration of 943.3 ± 177.4 and 952.5 ± 98.8 days (*P* = 0.683) for patients with negative and those with positive T-Spot.TB results, respectively, those with positive T-Spot.TB maintained a higher CD4 count (592.8 ± 294.9 vs 451.9 ± 274.3 cells/μL, *P*<0.001) and lower plasma HIV RNA viral load (1.85 ± 0.97 vs 2.64 ± 1.50 log_10_ copies/mL, *P*<0.001) than those with negative T-Spot.TB despite the fact that similar proportions of the two groups were on cART (92.2% vs 90.1%, *P* = 0.589).

**Table 1:** Demographic and clinical characteristics of HIV-infected patients with negative, positive, and indeterminate T-Spot.TB results.

Results of T-Spot.TB	Negative	Positive	Indeterminate	P^a^ value	P^b^ value
Patient No., n (%)	534 (87.8)	64 (10.5)	10 (1.6)		
Age, mean (SD)	36.9 (10.8)	45 (13.1)	41.1 (14.4)	<0.001	<0.001
Male gender, n (%)	521 (97.6)	58 (90.6)	10 (100.0)	0.023	0.009
Risk for HIV transmission, %					
Homosexuals	83.2	70.6	71.4	0.056	0.077
Heterosexuals	13.6	27.5	14.3		
Injecting drug users	1.4	2.0	0		
Others	1.8	0	14.3		
At T-Spot.TB testing, n (%)					
Previous TB, n (%)	20 (3.7)	24 (37.5)	0 (0)	<0.001	<0.001
CD4 lymphocyte count, mean (SD), cells/μl	441.6 (295.9)	578.4 (306.5)	473.5 (316.0)	<0.001	0.001
CD4 <200, n (%)	93 (18.5)	10 (14.5)	1 (11.1)	0.200	0.422
PVL, mean (SD), log_10_ copies/mL	2.89 (1.50)	2.35 (1.23)	2.79 (1.84)	0.005	0.003
PVL >5 log_10_ copies/mL, n (%)	59 (11.8)	1 (1.7)	1 (11.1)	0.029	0.019
PVL <50 copies/mL, n (%)	223 (44.5)	37 (63.8)	6 (66.7)	0.009	0.005
Newly diagnosed HIV infection, n (%)	55 (10.4)	4 (6.3)	1 (10.0)	0.36	0.315
On cART, n (%)	326 (61.0)	54 (84.4)	6 (60.0)	0.001	<0.001
End of follow-up					
CD4 lymphocyte count, mean (SD), cells/μl	451.9 (274.3)	592.8 (294.9)	429.9 (319.3)	0.003	<0.001
CD4 <200, n (%)	87 (16.7)	4 (6.3)	3 (30.0)	0.031	0.030
PVL, mean (SD), log_10_ copies/mL	2.64 (1.50)	1.85 (0.97)	1.94 (1.42)	<0.001	<0.001
PVL >5 log_10_ copies/mL, n (%)	48 (9.2)	1 (1.6)	1 (10.0)	0.069	0.037
PVL <50 copies/mL, n (%)	251 (48.2)	46 (71.9)	7 (70.0)	<0.001	<0.001
On cART, n (%)	481 (90.1)	59 (92.2)	10 (100)	0.739	0.589
Duration of follow-up, mean (SD), person-days	943.3 (177.4)	952.5 (98.8)	868.8 (392.7)	0.586	0.683
Loss to follow-up, n (%)	66 (12.4)	6 (9.4)	0 (0)	0.647	0.488
Death, n (%)	13 (2.4)	0 (0)	2 (20.0)	0.048	0.464

Note:

^a^ Comparison among patients with negative, positive, and indeterminate T-Spot.TB results

^b^ Comparison between patients with negative and positive T-Spot.TB results

**Abbreviations:** cART, combination antiretroviral therapy; PVL, plasma HIV RNA load; SD, standard deviation; TB, tuberculosis

In multivariate analysis, we included variables with significant difference between the two groups in univariate analysis (Table 1), such as age, male gender, a history of old TB, CD4 count, plasma HIV RNA load at enrollment and cART use, and decided the final model by using the Hosmer-Lemeshow goodness-of-fit test. We found that the independent factors associated with positive T-Spot.TB results were older age (adjusted odds ratio [AOR], per 10-year increase, 1.172; 95% confidence interval [CI], 1.022–1.344, *P* = 0.023), past history of TB (AOR, 13.412; 95% CI, 6.106–29.460, *P*<0.001), and higher CD4 counts at enrollment (AOR, per 50-cell/μl increase, 1.062; 95% CI, 1.017–1.109, *P* = 0.007) (Table 2).

**Table 2:** Multivariate analysis for factors associated with positive T-Spot.TB results.

Variables	Reference	Odds ratio	95% confidence interval	*P* value
Age (per 10-year increase)	Continuous variable	1.172	1.022–1.344	0.023
Male	Female	0.399	0.104–1.533	0.181
Old TB	No old TB	13.412	6.106–29.460	<0.001
CD4 counts at enrollment (per 50-cell/μl increase)	Continuous variable	1.062	1.017–1.109	0.007
Plasma HIV RNA load at enrollment (per 1-log_10_ increase)	Continuous variable	0.804	0.629–1.028	0.082

**Abbreviations:** PVL, plasma HIV RNA load; TB, tuberculosis

Of the 64 patients with positive T-Spot.TB, 24 patients (37.5%) had a history of old TB with a mean interval of 2556 days (range, 209 to 5187 days) between T-Spot.TB testing and reporting date of TB. None of the 64 patients received IPT; all but 5 patients continued to receive cART at the end of follow-up. The latest mean CD4 count and plasma HIV RNA viral load at the end of follow-up of the 64 patients were 592.8 cells/μL and 1.85 log_10_ copies/mL, respectively. A diagnosis of active TB was made in 1 patient (1.6%) after a follow-up of 337 days who was receiving cART with a CD4 count of 78 cells/μL and plasma HIV viral load of 98 copies/mL. The incidence rate of TB in these 64 patients with positive result was 0.599 per 100 PYFU after a total follow-up of 167 person-years, and 0.064 per 100 PYFU in 608 enrolled patients without active TB at baseline after a total follow-up duration of 1571 person-years. None of the 24 patients with previous TB who tested positive for T-Spot.TB developed active or recurrent TB after a total follow-up duration of 62.33 person-years. Thus, the positive predictive value for developing active TB in those testing positive for T-Spot.TB was only 1.6% (1/64).

Of 534 patients with negative T-Spot.TB results, 20 patients (3.7%) had a history of old TB with a mean interval of 3117 days (range, 141 to 6361 days) between T-Spot.TB testing and reporting date of TB. None of the 534 patients developed active TB after a total follow-up of 1380 person-years, and 481 (90.1%) of them received cART at the end of follow-up with the latest mean CD4 and plasma HIV RNA load of 451.9 cells/μL and 2.64 log_10_ copies/mL, respectively. Thus, the negative predictive value for developing active TB in those testing negative for T-Spot.TB was 100%.

Of the 10 patients with indeterminate T-Spot.TB, their median CD4 counts were 458.8 cells/μL (range, 0 to 934 cells/μL) and median plasma HIV RNA load 1.60 log_10_ copies/mL (range, 2 to 6 log_10_ copies/mL) at the time of T-Spot.TB testing; none had a history of TB. After a total follow-up of 23.8 person-years when all of the patients were on cART, none developed active TB with the latest median CD4 count of 438.6 cells/μL, and plasma HIV RNA load of 1.3 log_10_ copies/mL.

## Discussion

In this study conducted in a country of moderate TB endemicity, we found that the prevalence of latent TB infection diagnosed by positive T-Spot.TB was 10.5% (64/608) in HIV-infected patients. One of the 64 patients with positive T-Spot.TB and without IPT developed active TB during follow-up, yielding a positive predictive value of TB progression of 1.6%. The incidence rate of active TB was 0.598 per 100 PYFU in 64 patients with positive T-Spot.TB and 0.064 per 100 PYFU in 608 patients without active TB at baseline. None of the patients with negative T-Spot.TB developed active TB, which resulted in a negative predictive value of 100%.

The incidence rate and crude incidence of active TB in HIV-infected patients with positive IGRA results range from 0 to 4.621 cases per 100 person-years and from 0 to 20%, respectively (summarized in Table 3) [12–16], depending on the TB epidemiology of the country where the study was conducted, number of study subjects enrolled (range, 50 to 909 subjects), follow-up duration (range, 1.28 to 2.97 years), methods employed to detect and diagnose TB, receipt of IPT, CD4 counts (range, median, 305 to 447 cells/μL) and plasma HIV RNA loads (range, 1.85 to 4.8 log_10_ copies/mL), and the proportion of study subjects on cART (range, 0 to 100%).

**Table 3:** Comparisons of progression of tuberculosis in HIV-infected patients with interferon-gamma release assay testing.

Reference	Country	IGRA	^1^Patient No.	Positive IGRA, n (%)	Indeterminate IGRA, n (%)	Follow-up duration	No. of ^2^TB cases; incidence (%); ^3^incidence rate (cases/100 PY)	IPT	Median CD4, cells/uL	Median PVL, log_10_ copies/mL	cART, %
Presentstudy	Taiwan	T-Spot.TB	609	64 (10.5)	10 (1.6)	2.57 years	1; 1.6; 0.598	No	437	1.85	63.5
[13]	Taiwan	T-Spot.TB	909	134 (14.7)	0 (0)	2.97 years	^4^4; ^4^4.7; ^4^1.72	^5^Yes/No	447	3.25	40.6
[16]	China	T-Spot.TB	50	21 (42)	NA	36 months	0; 0; 0	No	305	NA	100
[15]	Korea	T-Spot.TB	120	30 (25)	7 (5.8)	32 months	6; 20; 4.621	No	306	NA	36.7
[14]	Kenya	T-Spot.TB	333	120 (36.4)	52 (15.6)	1.28 years	6; 5; 4.2	No	440	4.8	0
[12]	Austria	GFT-GIT	822	37 (4.5)	47 (5.7)	19 months	3; 8.1; NA	No	393	NA	59.6

**Note:**

^1^Patient No.: patient number after exclusion of patients with active TB at enrollment;

^2^TB cases, cases of patients with positive IGRA results who developed TB during follow-up;

^3^ The incidence rate of patients with positive IGRA results who developed TB during follow-up;

^4^Individuals with isoniazid prophylaxis therapy were excluded;

^5^Yes/No: 4 of 32 patients with positive IGRA results receiving no IPT developed TB and 1 of 49 patients with positive IGRA results receiving IPT developed TB;

**Abbreviations:** cART: combination antiretroviral therapy; IGRA, interferon-gamma release assay; IPT: isoniazid preventive therapy; NA: not available; PY, person-years; TB, tuberculosis

The incidence rate or crude rate in our patients with positive IGRA results appears to be lower than those reported in studies conducted in countries of different levels of TB endemicity (Table 3) [12–16, 18]. A recent study conducted in Brazil (TB incidence of 46 per 100,000 population [19]) reported a TB incidence of 6.52 cases per 100 PYFU in HIV-infected patients with positive TST and without IPT [18]. Compared with the study in Brazil [18], our study cohort had a higher cART coverage (84.4% vs. 53%), higher CD4 counts at enrollment (578.4 vs. 447 cells/uL) and a shorter follow-up duration (2.6 vs. 4.7 years), which may contribute to the observed difference in the incidence rate of active TB.

In the study conducted in Taiwan in which 40% of the subjects were incarcerated injecting drug users [13], Yang et al found an overall rate of 4.7% and an incidence rate of 1.72 per 100 PYFU of active TB in 909 subjects enrolled despite the fact that 87 of 227 patients (38.3%) with positive TST received IPT. Other than the differences in the composition and HIV disease status of the study subjects, the background TB incidence may also play a role in the development of active TB. Our study and that by Yang et al. were conducted over two different study periods (2011–2013 vs. 2008–2010), during which time the national TB incidence has decreased from 73 in 2005 to 53 per 100, 000 population in 2012 with the implementation of the intensified national TB programs launched in 2006 to halve the TB incidence by 2015 [17].

While the incidence rate of our HIV-infected patients testing positive for T-Spot.TB is lower than that reported in many other studies (Table 3), the rate (0.598 per 100 PYFU) remains significantly higher as compared to that in the general population despite free access to cART and sustained improvement of immunity with cART in our patients. To further decrease the risk of progression to TB among HIV-infected patients in Taiwan, earlier initiation of cART and IPT are the two strategies that have been confirmed to be beneficial in several clinical trials [20–23]. However, a substantial proportion of our HIV-infected patients presented late for HIV care [24, 25]. More efforts are needed to facilitate early diagnosis of HIV infection and to overcome the obstacles to linkage to and retention in HIV care, prescription of cART and achievement of sustained suppression of HIV replication [26].

Identification of latent TB infection and implementation of IPT has been recommended to achieve sustained decreases of TB incidence by an expert panel of review of the national TB program in Taiwan [17]. Such a recommendation could also be applied to HIV-infected patients as a randomized double-blind placebo-controlled trial that has demonstrated that all patients receiving antiretroviral therapy in moderate or high incidence areas should receive IPT regardless of the status of TST or IGRAs [23]. While concerns about the durability of IPT remains [27, 28], a recent Brazilian study demonstrates that the benefit of IPT may last 7 years in the cART era in a country with moderate TB endemicity [18].

Several limitations of our study deserve to be acknowledged. First, 80.9% of the study subjects were male homosexuals, and only 1.8% were injecting drug users. Our results may not be generalizable to injecting drug users with HIV infection who have been identified as a high-risk group for active TB [29]. Second, not all patients received regular work-up for TB during follow-up, though when the clinical index of suspicion was high, patients underwent extensive TB work-up. Third, 12.3% of the patients with positive IGRA results were lost to follow-up. Nevertheless, as we had access to the national TB registry and TB is a mandatory notifiable disease, data regarding the development of TB in those lost to follow-up were available. Fourth, we were not able to assess the risk factors for TB progression in those with positive IGRA results because only one patient developed TB during follow-up. Fifth, the follow-up duration in our study remains short. With ageing and increasing rates of morbidities in patients with prolonged exposure to cART in Taiwan [30], the risk for developing active TB warrants further investigation. Lastly, the study was conducted in a country of moderate TB endemicity and the results may not be generalizable to countries of higher TB endemicity where cART program may not be widely implemented.

In conclusion, the risk of developing active TB in HIV-infected patients positive for T-Spot.TB receiving cART is low in a country of moderate endemicity of TB as compared with that in other countries of similar endemicity.

## Supporting Information

S1 Data Raw data of HIV-infected patients testing for T-SPOT.TB. (XLS)

## References

[1] Comstock GW, Livesay VT, Woolpert SF. 1974 The prognosis of a positive tuberculin reaction in childhood and adolescence. American journal of epidemiology 99:131–8. 4810628 10.1093/oxfordjournals.aje.a121593

[2] Small PM, Fujiwara PI. 2001 Management of tuberculosis in the United States. N Engl J Med 345:189–200. 11463015 10.1056/NEJM200107193450307

[3] Rothel JS, Jones SL, Corner LA, Cox JC, Wood PR. 1990 A sandwich enzyme immunoassay for bovine interferon-gamma and its use for the detection of tuberculosis in cattle. Australian veterinary journal 67:134–7. 2115767 10.1111/j.1751-0813.1990.tb07730.x

[4] Converse PJ, Jones SL, Astemborski J, Vlahov D, Graham NM. 1997 Comparison of a tuberculin interferon-gamma assay with the tuberculin skin test in high-risk adults: effect of human immunodeficiency virus infection. The Journal of infectious diseases 176:144–50. 9207360 10.1086/514016

[5] Streeton JA, Desem N, Jones SL. 1998 Sensitivity and specificity of a gamma interferon blood test for tuberculosis infection. The international journal of tuberculosis and lung disease: the official journal of the International Union against Tuberculosis and Lung Disease 2:443–50. 9626600

[6] Mazurek GH, Jereb J, Vernon A, LoBue P, Goldberg S, Castro K, et al 2010 Updated guidelines for using Interferon Gamma Release Assays to detect Mycobacterium tuberculosis infection—United States, 2010. MMWR. Recommendations and reports: Morbidity and mortality weekly report. Recommendations and reports / Centers for Disease Control 59:1–25. 20577159

[7] Pai M, Zwerling A, Menzies D. 2008 Systematic review: T-cell-based assays for the diagnosis of latent tuberculosis infection: an update. Annals of internal medicine 149:177–84. 18593687 10.7326/0003-4819-149-3-200808050-00241 PMC2951987

[8] Cattamanchi A, Smith R, Steingart KR, Metcalfe JZ, Date A, Coleman C, et al 2011 Interferon-gamma release assays for the diagnosis of latent tuberculosis infection in HIV-infected individuals: a systematic review and meta-analysis. Journal of acquired immune deficiency syndromes 56:230–8. 10.1097/QAI.0b013e31820b07ab 21239993 PMC3383328

[9] Santin M, Munoz L, Rigau D. 2012 Interferon-gamma release assays for the diagnosis of tuberculosis and tuberculosis infection in HIV-infected adults: a systematic review and meta-analysis. PloS one 7:e32482 10.1371/journal.pone.0032482 22403663 PMC3293815

[10] Rangaka MX, Wilkinson KA, Glynn JR, Ling D, Menzies D, Mwansa-Kambafwile J, et al 2012 Predictive value of interferon-gamma release assays for incident active tuberculosis: a systematic review and meta-analysis. The Lancet. Infectious diseases 12:45–55. 10.1016/S1473-3099(11)70210-9 21846592 PMC3568693

[11] Diel R, Loddenkemper R, Nienhaus A. 2012 Predictive value of interferon-gamma release assays and tuberculin skin testing for progression from latent TB infection to disease state: a meta-analysis. Chest 142:63–75. 10.1378/chest.11-3157 22490872

[12] Aichelburg MC, Rieger A, Breitenecker F, Pfistershammer K, Tittes J, Eltz S, et al 2009 Detection and prediction of active tuberculosis disease by a whole-blood interferon-gamma release assay in HIV-1-infected individuals. Clinical infectious diseases: an official publication of the Infectious Diseases Society of America 48:954–62. 19245343 10.1086/597351

[13] Yang CH, Chan PC, Liao ST, Cheng SH, Wong WW, Huang LM, et al 2013 Strategy to better select HIV-infected individuals for latent TB treatment in BCG-vaccinated population. PloS one 8:e73069 10.1371/journal.pone.0073069 24015285 PMC3754919

[14] Jonnalagadda S, Lohman Payne B, Brown E, Wamalwa D, Maleche Obimbo E, Majiwa M, et al 2010 Latent tuberculosis detection by interferon gamma release assay during pregnancy predicts active tuberculosis and mortality in human immunodeficiency virus type 1-infected women and their children. The Journal of infectious diseases 202:1826–35. 10.1086/657411 21067370 PMC3058232

[15] Kim YJ, Kim SI, Kim YR, Wie SH, Park YJ, Kang MW. 2012 Predictive value of interferon-gamma ELISPOT assay in HIV 1-infected patients in an intermediate tuberculosis-endemic area. AIDS research and human retroviruses 28:1038–43. 10.1089/AID.2011.0360 22352311

[16] Zhang LF, Liu XQ, Zuo LY, Li TS, Deng GH, Wang AX. 2010 Longitudinal observation of an interferon gamma-released assay (T-SPOT.TB) for Mycobacterium tuberculosis infection in AIDS patients on highly active antiretroviral therapy. Chinese medical journal 123:1117–21. 20529548

[17] Chan PC, Chen CH, Chang FY. 2014 External review of the National Tuberculosis Program and the development of strategy and targets post 2015 in Taiwan. Journal of the Formosan Medical Association = Taiwan yi zhi 113:775–7. 10.1016/j.jfma.2014.03.003 24787665

[18] Golub JE, Cohn S, Saraceni V, Cavalcante SC, Pacheco AG, Moulton LH, et al 2014 Long-term Protection From Isoniazid Preventive Therapy for Tuberculosis in HIV-Infected Patients in a Medium-Burden Tuberculosis Setting: The TB/HIV in Rio (THRio) Study. Clinical infectious diseases: an official publication of the Infectious Diseases Society of America. 10.1093/cid/ciu849 PMC4366579 25365974

[19] World Health Organization 2013, posting date. Global Tuberculosis Report 2013. [Online.]

[20] Suthar AB, Lawn SD, del Amo J, Getahun H, Dye C, Sculier D, et al 2012 Antiretroviral therapy for prevention of tuberculosis in adults with HIV: a systematic review and meta-analysis. PLoS medicine 9:e1001270 10.1371/journal.pmed.1001270 22911011 PMC3404110

[21] Collins SE, Jean Juste MA, Koenig SP, Secours R, Ocheretina O, Bernard D, et al 2015 CD4 deficit and tuberculosis risk persist with delayed antiretroviral therapy: 5-year data from CIPRA HT-001. The international journal of tuberculosis and lung disease: the official journal of the International Union against Tuberculosis and Lung Disease 19:50–7. 10.5588/ijtld.14.0217 25519790

[22] Grinsztejn B, Hosseinipour MC, Ribaudo HJ, Swindells S, Eron J, Chen YQ, et al 2014 Effects of early versus delayed initiation of antiretroviral treatment on clinical outcomes of HIV-1 infection: results from the phase 3 HPTN 052 randomised controlled trial. The Lancet. Infectious diseases 14:281–90. 10.1016/S1473-3099(13)70692-3 24602844 PMC4144040

[23] Rangaka MX, Wilkinson RJ, Boulle A, Glynn JR, Fielding K, van Cutsem G, et al 2014 Isoniazid plus antiretroviral therapy to prevent tuberculosis: a randomised double-blind, placebo-controlled trial. Lancet 384:682–90. 10.1016/S0140-6736(14)60162-8 24835842 PMC4233253

[24] Hung CC, Hsiao CF, Chen MY, Hsieh SM, Chang SY, Sheng WH, et al 2006 Improved survival of persons with human immunodeficiency virus type 1 infection in the era of highly active antiretroviral therapy in Taiwan. Japanese journal of infectious diseases 59:222–8. 16936339

[25] Lo YC, Wu PY, Hsieh CY, Chen MY, Sheng WH, Hsieh SM, et al 2011 Late diagnosis of human immunodeficiency virus infection in the era of highly active antiretroviral therapy: role of socio-behavioral factors and medical encounters. Journal of the Formosan Medical Association = Taiwan yi zhi 110:306–15. 10.1016/S0929-6646(11)60046-6 21621151

[26] Hall HI, Frazier EL, Rhodes P, Holtgrave DR, Furlow-Parmley C, Tang T, et al 2013 Differences in human immunodeficiency virus care and treatment among subpopulations in the United States. JAMA internal medicine 173:1337–44. 10.1001/jamainternmed.2013.6841 23780395

[27] Churchyard GJ, Fielding KL, Grant AD. 2014 A trial of mass isoniazid preventive therapy for tuberculosis control. N Engl J Med 370:1662–3. 10.1056/NEJMc1402073 24758626

[28] Samandari T, Agizew TB, Nyirenda S, Tedla Z, Sibanda T, Shang N, et al 2011 6-month versus 36-month isoniazid preventive treatment for tuberculosis in adults with HIV infection in Botswana: a randomised, double-blind, placebo-controlled trial. Lancet 377:1588–98. 10.1016/S0140-6736(11)60204-3 21492926

[29] van Asten L, Langendam M, Zangerle R, Hernandez Aguado I, Boufassa F, Schiffer V, et al 2003 Tuberculosis risk varies with the duration of HIV infection: a prospective study of European drug users with known date of HIV seroconversion. Aids 17:1201–8. 12819522 10.1097/00002030-200305230-00012

[30] Wu PY, Chen MY, Hsieh SM, Sun HY, Tsai MS, Lee KY, et al 2014 Comorbidities among the HIV-infected patients aged 40 years or older in Taiwan. PloS one 9:e104945 10.1371/journal.pone.0104945 25119532 PMC4132082

